# The Association between Low Blood Pressure and Attention-Deficit Hyperactivity Disorder (ADHD) Observed in Children/Adolescents Does Not Persist into Young Adulthood. A Population-Based Ten-Year Follow-Up Study

**DOI:** 10.3390/ijerph18041864

**Published:** 2021-02-14

**Authors:** Jan Schulz, Franziska Huber, Robert Schlack, Heike Hölling, Ulrike Ravens-Sieberer, Thomas Meyer, Luise Poustka, Aribert Rothenberger, Biyao Wang, Andreas Becker

**Affiliations:** 1 Department of Child and Adolescent Psychiatry and Psychotherapy, University Medical Center Göttingen, 37075 Göttingen, Germany; jan.schulz@med.uni-goettingen.de (J.S.); franziska.huber1@stud.uni-goettingen.de (F.H.); luise.poustka@med.uni-goettingen.de (L.P.); arothen@gwdg.de (A.R.); abecker4@gwdg.de (A.B.); 2 Robert Koch Institute, Department of Epidemiology and Health Monitoring, Unit Mental Health, 13353 Berlin, Germany; SchlackR@rki.de (R.S.); HoellingH@rki.de (H.H.); 3 Department of Child and Adolescent Psychiatry, Psychotherapy and Psychosomatics, University Medical Center Hamburg-Eppendorf, 20246 Hamburg, Germany; ravens-sieberer@uke.de; 4 Department of Psychosomatic Medicine and Psychotherapy, University Medical Center Göttingen, 37075 Göttingen, Germany; thomas.meyer@med.uni-goettingen.de; 5 German Centre for Cardiovascular Research, Partner Site Göttingen, 10115 Berlin, Germany; 6 Department of Clinical, Educational and Health Psychology, University College London, London WC1H 0AP, UK

**Keywords:** ADHD, KiGGS study, arousal, blood pressure, long-term changes, children, young adults

## Abstract

*Background*: Attention-deficit hyperactivity disorder (ADHD) is one of the most common behavioral disorders in childhood and adolescence associated with relevant psychosocial impairments. The basic pathophysiology of ADHD may be related, at least partly, to a deficit in autonomic arousal processes, which not only influence core symptoms of the disorder, but may also lead to blood pressure (BP) deviations due to altered arousal regulation. *Objectives*: This study examined long-term changes in BP in children and adolescents with ADHD up to young adulthood. Methods: In children and adolescents aged between 7 and 17 years at baseline, we compared BP recordings in subjects with (*n* = 1219, 11.1%) and without (*n* = 9741, 88.9%) ADHD over a 10-year follow-up using data from the nationwide German Health Survey for Children and Adolescents (KiGGS). Propensity score matching was used to improve the comparability between children in the ADHD and control groups with now *n* = 1.190 in each group. *Results*: The results of these matched samples revealed that study participants with ADHD showed significantly lower systolic BP (107.6 ± 10.7 mmHg vs. 109.5 ± 10.9 mmHg, *p* < 0.001, Cohen’s d = 0.17) and diastolic BP (64.6 ± 7.5 mmHg vs. 65.8 ± 7.4 mmHg, *p* < 0.001, Cohen’s d = 0.16) at baseline. In a sensitivity analysis with a smaller (*n* = 272) and more stringently diagnosed ADHD group, the significant differences remained stable with somewhat higher Cohen’s d; i.e., 0.25 and 0.27, respectively. However, these differences did not persist after 10-year follow-up in a smaller matched longitudinal sub-group (ADHD *n* = 273; control *n* = 323), as subjects with and without ADHD had similar levels of systolic (123.4 ± 10.65 vs. 123.78 ± 11.1 mmHg, *p* = 0.675, Cohen’s d = 0.15) and diastolic BP (71.86 ± 6.84 vs. 71.85 ± 7.06 mmHg, *p* = 0.992, Cohen’s d = 0.16). *Conclusions:* At baseline, children and adolescents with ADHD had significantly lower BP (of small effect sizes) compared to the non-ADHD group, whereas this difference was no longer detectable at follow-up ten years later. These developmental alterations in BP from adolescence to early adulthood may reflect changes in the state of autonomic arousal, probably modulating the pathophysiology of ADHD.

## 1. Introduction

Attention-deficit hyperactivity disorder (ADHD) is one of the most common behavioral disorders of childhood and adolescence, affecting 5% of school children and 3% of adults [1,2,3]. Inattentiveness, hyperactivity, and impulsiveness that deviate from developmental age are typical symptoms of the disorder [4]. In addition to the core symptomatology, comorbid psychiatric disorders can lead to long-term social, educational, and professional impairments [5]. Genetic factors and early environmental risks, which interact in a complex way and influence structural and functional brain development, play an essential role and probably contribute to the high etiological heterogeneity in this neurodevelopmental disorder. In ADHD patients, a total volume reduction of the brain [6,7] as well as volume reductions in prefrontal areas, the basal ganglia, and the cerebellum have been described [8,9,10,11]. It can be assumed that these structural changes are related to physiological processes within different cortical as well as subcortical brain circuits and are responsible for both the profile of clinical symptoms and severity of the disorder [8,12]. Specifically, this study focuses on the subcortical circuits of the autonomic nervous system. For example, structural brainstem abnormalities of the substantia nigra in ADHD and related deviations of autonomic sleep parameters [13] seem to play a role and thus support the arousal hypothesis in ADHD [14,15,16], including low subcortical functional activation. The autonomic nervous system has a fundamental impact on the physiological and psychological regulation of attention and alertness, which is called arousal [17,18,19,20,21]. The degree of arousal is controlled by complex interactions between the peripheral and central nervous system. The latter is reflected in activation patterns of high frequency cortical oscillations like the theta/beta ratio [22]. From an evolutionary point of view, the ability to continuously adapt the level of arousal to changing environmental conditions/situations represents a survival advantage. Different psychiatric disorders can be accompanied by an impaired arousal regulation. For example, an excessively stable arousal state has been described in depression [23], whereas unstable arousal states have been observed in bipolar mania and ADHD [24,25].

Various studies have shown that in ADHD both the peripheral arousal and the cortical arousal are decreased and unstable [26,27,28]. However, it is unclear whether disturbed arousal could be responsible for both impaired blood pressure (BP) regulation and reactive, compensatory autoregulation of behavior resulting in hyperactivity and sensation seeking. Negrao et al. investigated heart rate variability and skin conductance level in children with ADHD, in the presence and absence of stimulant therapy [29]. It was shown that, without adequate therapy, there was an overactivation of the parasympathetic nervous system and an underactivation of the sympathetic nervous system. Treatment with methylphenidate could partially normalize the autonomic imbalance. Investigations on the diurnal variations of the arousal (measured with heart rate and cortisol) in children with ADHD speak in favor of such an autonomous imbalance (probably closely connected with a dysregulation of the locus coeruleus and the reticular network). On the basis of cortisol measurements in saliva and Holter monitorings of heart rate, not only an overall increased heart rate was found in ADHD, but also a morning hypo-arousal and an afternoon/night hyper-arousal in circadian analysis [30,31,32,33].

Though a multifactorial etiology is suspected in ADHD [34], in the literature there are numerous references to an arousal dysregulation, which seems to partly cause or modulate the symptom constellation in ADHD. Autostimulative overcompensation of an underlying hypoarousal was described as a compensatory attempt to stabilize brain arousal in ADHD, which may contribute to the ADHD core symptomatology [25]. Dietrich et al. reported relationships between peripheral BP regulation, arousal, and cognitive performance in ADHD [35]. In untreated children with ADHD, usually associated with low BP, this mechanism could therefore be involved in the psychosocial problems linked to this disorder [36]. However, since the existing data on BP regulation in ADHD patients (as a reflection of autonomic arousal) are scarce and inconclusive, the aim of this study was to investigate the assumption of a lower BP in ADHD further.

Using the baseline data of the epidemiological, community-based, observational German Health Interview and Examination Survey for Children and Adolescents (KiGGS) survey, we previously reported a significantly lower systolic, diastolic, and mean arterial BP in children (11–17 years) diagnosed with ADHD as compared to a control group [37]. Furthermore, we demonstrated that the severity of ADHD symptoms was associated with lower BP recordings in both univariate and multivariate analyses. However, whether this relationship can be seen also in younger children with ADHD and whether it persists in young adulthood as time goes by remains open. Using data from the second wave of the KiGGS study, we tested the hypothesis that lower BP could also be observed longitudinally in adolescents and young adults with ADHD after a ten-year interval.

## 2. Materials and Methods

### 2.1. KiGGS Study Design

Our analysis is based on data from the KiGGS study, a nationwide and representative survey conducted by the Robert Koch Institute, Berlin, Germany, in several waves. Between 2003 and 2006, representative data on the health status of children and adolescents aged between 0 and 17 years were collected in the KiGGS baseline survey using a random sample of *n* = 17,641 participants in 167 cities and municipalities in Germany [38,39]. In the three-year study period, the children and adolescents (from the age of 11 years upwards) and also their parents were interviewed in writing, and computer-assisted medical interviews and physical examinations (including BP and heart rate) were conducted [40,41]. KiGGS Wave 1 conducted between 2009 and 2013 also aimed to make statements on the individual physical, psychological, and social development of children and adolescents [41]. Since Wave 1 was a pure telephone interview-based data collection and no physical examinations were carried out, no values for BP and heart rate are available from this period. The data collection of KiGGS Wave 2 was carried out from 2014 to 2017. The first participants who had agreed to re-contact were invited to participate in the study again and were 10 to 31 years old at the time of data collection. The current study included participants aged from seven to 17 years at baseline, which resulted in a total sample of N = 10,960 children. The majority of participants had physical measures of BP at baseline (*n* = 10,937). A total of 10,853 subjects from the baseline survey cohort also participated in Wave 2, corresponding to 61.5% of the participants in the baseline study. In Wave 2, physical examinations were again performed, in addition to written and oral interviews [42]. For the cohort analysis, BP and heart rate values were recorded for the second time as follow-up in Wave 2. Physical examination was offered only to those participants who still lived in the place where they first participated. In the case of non-participation, only a survey was requested [43]. Due to this circumstance, only about one fourth of the participants had physical measurements of BP at the ten-year follow up (*n* = 2907).

**Figure 1:**
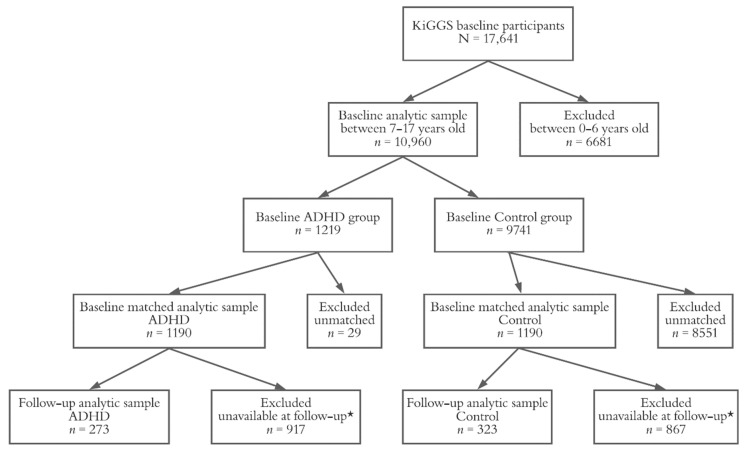
Group participants and matching flowchart. Note: * Participants were excluded due to lack of physical measurements of blood pressure at the ten-year follow up.

The group-related design and flowchart of our study can be seen in Figure 1.

### 2.2. Assessment of ADHD Symptoms

Participants were included in the group of confirmed ADHD cases if a corresponding pre-diagnosis, at that time in Germany on the basis of the International Classification of Diseases 10 (ICD-10)/Diagnostic and Statistical Manual of Mental Disorders 4 (DSM-4)), given by either a physician or psychologist, had been reported by the parents at the beginning of the study inclusion (*n* = 603, 5.5%). Participants with a score of equal to or greater than seven on the hyperactivity/inattention subscale of the Strengths and Difficulties Questionnaire (SDQ) were classified as suspected cases (*n* = 888, 8.10%) [36,38,44]. The SDQ is a short questionnaire covering the most important areas of psychopathology in children and adolescents. It consists of 25 items, which can be assigned to five subscales: emotional problems, behavioral problems, hyperactivity, peer problems, and prosocial behavior [45,46]. The SDQ dysregulation profile describes a combination of SDQ items in terms of a broader, dimensional, psychopathological concept [47]. In our analysis, study participants with diagnosed ADHD or suspected ADHD (SDQ hyperactivity/inattention subscale score ≥ 7) were combined in the ADHD risk group (*n* = 1219, 11.1%, including *n* = 272 children diagnosed with both ADHD and SDQ ≥ 7), while *n* = 9741 (88.9%) subjects were considered as non-ADHD controls.

### 2.3. Blood Pressure and Heart Rate Measurements

In all participants in the KiGGS study aged between three and 17 years, systolic and diastolic BP recordings, as well as arterial mean pressure and heart rate were determined at two recordings by means of an oscillometric measurement using an automatic, digital BP monitor (Datascope Accutorr Plus, Mindray DS, Mahwah, NJ, USA). The measurements were performed on the right arm, the subject in a seated position, having rested for five and seven minutes, respectively. The right elbow was positioned at the level of the heart and a BP cuff was selected which covered two thirds of the unclothed upper arm of the proband [48].

### 2.4. Statistical Analysis

Demographic characteristics as well as laboratory measurements of eligible participants were firstly examined in a descriptive manner with numerical data expressed as means and standard deviations, and categorical data expressed as frequencies and percentages. The differences between individuals in the ADHD and control group were compared using chi-square tests for categorical variables and *t*-tests for continuous variables. Propensity score matching was used to improve the comparability between the ADHD and control group. The propensity score matching approximates the counterfactual scenario by creating statistically matched groups of individuals that differ solely in their classification to the ADHD group, while the observed covariates/confounders were kept similar [49]. The propensity score for each individual was generated through a model predicting the risk of classification to the ADHD group, and this score was then used to match the two groups. The exposed group was matched with varying numbers of controls, where the smaller the number (e.g., one-to-one matching), the smaller the propensity-score distance and less bias. In this analysis, we used the commonly employed matching method of the nearest neighbor matching, where for each participant in the ADHD group, the participant with the closest propensity score (i.e., the smallest distance) was found in the control group, while the non-selected controls were discarded. The distributions of ADHD propensity for individuals in the ADHD and the control group before and after matching were presented in bar charts. After the comparable ADHD and control groups were established (herein referred as the matched sample), we tested the predictive value of BP indicators on ADHD using logistic regression models. For each potential predictor, including the BP indicators (systolic, diastolic, and pulse pressure) as well as demographic (age, sex, socioeconomic status) and laboratory measurements (body-mass index (BMI), heart rate), a univariate logistic regression (i.e., the unadjusted model) was conducted to test whether it predicted ADHD (i.e., individual’s group membership in the ADHD vs. control groups). For each BP indicator, a multivariate logistic regression model was created, the demographic and laboratory measurements (i.e., the adjusted model) being controlled. Individuals in the matched sample were followed over ten years (herein referred as the followed-and-matched sample). Descriptive comparisons on measurements at baseline, follow-up, as well as changes from baseline to follow-up were reported. Multivariate logistic regressions were conducted in the followed-and-matched sample to test the predictive value of BP indicators at (a) baseline to test the sensitivity of the relationship due to sample shrinkage and (b) follow-up to study the persistence of the relationship in the course of a longitudinal development. All statistical analyses were performed in R version 3.6.1 [50] and statistical significance was defined as *p* < 0.05 in all tests. Effect sizes were calculated in the form of Cohen’s d for *t*-tests, Cohen’s ω for chi-square tests, and odds ratios/Exp (ß) for logistic regressions. As sensitivity analysis, we compared participants in the control group and participants with more stringent ADHD, namely who fulfilled both criteria of pre-diagnosed ADHD and SDQ-H ≥ 7, to strengthen our findings (see addendum in Supplementary Material).

## 3. Results

### 3.1. Characterization of the KiGGS Baseline Cohort

**Figure 2:**
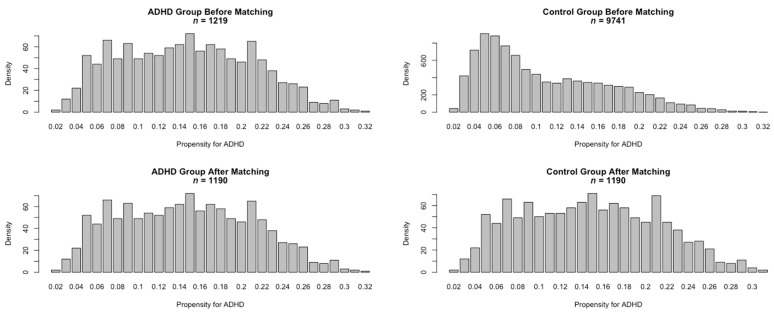
Distribution of the propensity score for attention-deficit hyperactivity disorder (ADHD) (before and after matching).

Among the total KiGGS study cohort aged 7 to 17 years (N = 10,960), there were *n* = 603 (5.5%) participants diagnosed as suffering from ADHD by a clinician or psychologist, and *n* = 888 (8.1%) participants fell into the suspected ADHD category with their score from the SDQ hyperactivity-inattention subscale ≥ 7. The diagnosed ADHD and suspected ADHD group together consisted of the ADHD risk group in the current study (*n* = 1219, 11.1%, including *n* = 272 children with both diagnosed and suspected ADHD), while the rest of the participants (*n* = 9741, 88.9%) were considered as the control group. As shown in the left panel of Table 1, compared with participants from the control group, those in the ADHD group were younger (*t*(10958) = −6.997, *p* < 0.001, Cohen’s d = 0.21), less likely to be female (*χ*^2^ (1, *N* = 10960) = 264.440, *p* < 0.001, Cohen’s ω = 0.16), from lower socioeconomic background (*t*(10651) = −9.528, *p* < 0.001, Cohen’s d = 0.29), have lower body-mass index (*t*(10908) = −2.676, *p* < 0.001, Cohen’s d = 0.08), and similar level of average heart rate (*t*(10905) = −0.408, *p* = 0.683, Cohen’s d = 0.01). The upper panel of Figure 2 presents the distribution of participants’ propensity scores of ADHD risks based on the indicators. The 9741 participants in the control group showed a skewed distribution compared to the more normally distributed 1219 participants from the ADHD group. To improve the comparability of the ADHD group and control group in observed confounders, participants in the ADHD and control group were compared using propensity score matching with the nearest neighbor matching technique. The matched sample included 1190 participants from the ADHD group and the same number of participants from the control group with the closest propensity of ADHD risks. As shown in the right panel of Table 1, after matching, participants in the ADHD group were similar to participants from the control group in age (*t*(2378) = −0.137, *p* = 0.891, Cohen’s d = 0.01), sex ratio (*χ*^2^ (1, *N* = 2380) = 0.002, *p* = 0.963, Cohen’s ω = 0.002), socioeconomic background (*t*(2378) = −0.108, *p* = 0.912, Cohen’s d = 0.004), body-mass index (*t*(2378) = −0.253, *p* = 0.800, Cohen’s d = 0.01), and heart rate (*t*(2378) = 0.575, *p* = 0.565, Cohen’s d = 0.02). The distributions of the participants’ propensity scores for ADHD risks after matching are presented in the bottom panel of Figure 2. The distribution of the control group after matching approached normality and was similar to the distribution of the ADHD group, showing that the propensity score matching corrected for the confounding factors to a large extent.

### 3.2. Blood Pressure in the KiGGS Baseline Cohort

In the KiGGS baseline cohort of 10,960 participants, systolic and diastolic BP, as well as the pulse pressure were all significantly lower among participants in the ADHD group as compared with those in the control group (before matching: for systolic BP, *t*(10935) = −8.349, *p* < 0.001, Cohen’s d = 0.25; for diastolic BP, *t*(10935) = −7.572, *p* < 0.001, Cohen’s d = 0.23; for pulse pressure, *t*(10935) = −4.533, *p* < 0.001, Cohen’s d = 0.14, Table 1 left panel; and after matching: for systolic BP, *t*(1190) = −4.267, *p* < 0.001, Cohen’s d = 0.17; for diastolic BP, *t*(1190) = −3.859, *p* < 0.001, Cohen’s d = 0.16; for pulse pressure, *t*(1190) = −2.192, *p* = 0.028, Cohen’s d = 0.09, Table 1 right panel). Results from univariate logistic regression analyses in the upper left panel of Table 2 showed that without adjustment for confounding variables, all hemodynamic parameters tested, namely systolic BP, diastolic BP, and pulse pressure, were significantly associated with ADHD. In the multivariate logistic models shown in the bottom left panel of Table 2, the associations between BP and ADHD were tested with age, sex, socioeconomic status, body-mass index, and heart rate controlled. The systolic BP (exp(ß) = 0.968, 95%-CI 0.961–0.975, *p* < 0.001), diastolic BP (exp(ß) = 0.969, 95%-CI 0.960–0.978, *p* < 0.001), and pulse pressure (exp(ß) = 0.983, 95%-CI 0.974–0.992, *p* < 0.001) were all independently and significantly associated with ADHD in separate models. In the matched sample of 2380 participants, the differences in systolic and diastolic BP, as well as the pulse pressure, among participants in the ADHD and control group were smaller but remained statistically significant (for systolic BP, exp(ß) = 0.984, 95%-CI 0.976–0.991, *p* < 0.001; for diastolic BP, exp(ß) = 0.979, 95%-CI 0.968–0.990, *p* < 0.001; for pulse pressure, exp(ß) = 0.989, 95%-CI 0.978–0.999, *p* = 0.029, Table 2 right panel). Similarly, the multivariate logistic regression showed consistent reverse associations between ADHD and the systolic BP (exp(ß) = 0.971, 95%-CI 0.961–0.981, *p* < 0.001), diastolic BP (exp(ß) = 0.973, 95%-CI 0.961–0.985, *p* < 0.001), and pulse pressure (exp(ß) = 0.985, 95%-CI 0.973–0.997, *p* = 0.016), whereas the majority of the confounders were no longer significantly associated with ADHD.

### 3.3. The Matched Sample at KiGGS Follow-Up

About one-fourth of participants (*n* = 596; aged 18–24 years) of the matched sample had BP measurements taken at KiGGS follow-up ten years later. The upper panel of Table 3 shows the 596 participants’ characteristics at baseline according to their classification into the ADHD or control group at baseline. The ADHD and control group remained to be comparable, reflected by the similar level of confounding variables at baseline, including age (*t*(594) = 0.153, *p* = 0.879, Cohen’s d = 0.01), sex ratio (*χ*^2^ (1, *N* = 596) = 0.610, *p* = 0.435, Cohen’s ω = 0.04), socioeconomic status (*t*(594) = 0.950, *p* = 0.342, Cohen’s d = 0.08), body-mass index (*t*(594) = 0.407, *p* = 0.684, Cohen’s d = 0.3), and heart rate (*t*(594) = 1.146, *p* = 0.252, Cohen’s d = 0.15). At follow-up, the differences in systolic and diastolic BP, as well as the pulse pressure among those participants in the ADHD and control group were no longer significant at the descriptive level (for systolic BP, t(594) = −1.811, *p* = 0.071, Cohen’s d = 0.15; for diastolic BP, t(594) = −1.927, *p* = 0.055, Cohen’s d = 0.16; for pulse pressure, t(594) = −0.646, *p* = 0.519, Cohen’s d = 0.05). In contrast, the baseline results of this reduced sample size (left panel of Table 4) showed that the associations between systolic BP (exp(ß) = 0.969, 95%-CI 0.949–0.991, *p* = 0.005) and diastolic BP (exp(ß) = 0.967, 95%-CI 0.942–0.992, *p* = 0.009), respectively, and ADHD were significant when the confounders were controlled for. Hence, the associations at baseline remained significant regardless of sample size shrinkage due to technical matching or follow-up measurement (*n* = 10,960 vs. *n* = 2380 vs. *n* = 596), suggesting the reverse relationships between ADHD and systolic or diastolic BP, but not pulse pressure, at a younger age. The middle and bottom panels of Table 3 showed the measurements at follow-up as well as the difference between those measurements at baseline vs. follow-up. Body-mass index and BP experienced an increase during the ten-year period, while the heart rate decreased. Participants in the ADHD and control group had similar levels for all those measurements, except for a significantly elevated level and change in body-mass index after ten years for the ADHD group. As is shown in the right panel of Table 4 (i.e., follow-up), even after having been controlled for confounders in the multivariate logistic regression models, the associations between ADHD and systolic BP (exp(ß) = 0.992, 95%-CI 0.975–1.009, *p* = 0.337), diastolic BP (exp(ß) = 0.993, 95%-CI 0.968–1.018, *p* = 0.567) and pulse pressure (exp(ß) = 0.991, 95%-CI 0.968–1.014, *p* = 0.423) were all not significant. However, there might be a tendency to a somewhat higher heart rate in ADHD at follow-up.

### 3.4. Sensitivity Analysis with Stringent ADHD Criteria

In comparison to the calculation described in Section 3.1, in the sensitivity analysis (flowchart, see Figure S1) with more stringent ADHD criteria from the total cohort (N = 10,960), the ADHD suspected group (*n* = 947, 8.6%), which had either only an SDQ-H ≥ 7, or only an ADHD prediagnosis, was excluded and only the cases with both characteristics were considered (*n* = 272, 2.5%). The control group (*n* = 9741, 88.8%) remained the same. Again in this calculation, systolic and diastolic BP and pulse pressure were significantly lower in participants in the ADHD group compared with the control group at baseline (before matching: for systolic BP, t(9990) = 5.0016, *p* < 0.001, Cohen’s d = 0.31; for diastolic BP, t(9990) = 3.9492, *p* < 0. 001, Cohen’s d = 0.24; for pulse pressure, t(9990) = 3.2898, *p* = 0.001, Cohen’s d = 0.20 (Table S1 left panel)). After matching more comparable participants with stringent ADHD and controls, the group differences on BP measures remained despite the vast shrinkage in sample size (for systolic BP, t(527) = 2.9068, *p* = 0.003, Cohen’s d = 0.25; for diastolic BP, t(527) = 3.1451, *p* = 0.001, Cohen’s d = 0.27; for pulse pressure, t(527) = 1.1904, *p* = 0.234, Cohen’s d = 0.10 (Table S1 right panel)). Regarding distributions of the participants’ propensity scores for ADHD risks before and after matching, see Supplementary Figure S1. In the matched sample of 530 participants, the differences in systolic and diastolic BP among participants in the ADHD and control group remained stable (for systolic BP, exp(ß) = 0.977 95%-CI 0.962–0.993, *p* = 0.004; for diastolic BP, exp(ß) = 0.963, 95%-CI 0.939–0.986, *p* = 0.002; for pulse pressure, exp(ß) = 0.987, 95%-CI 0.966–1.008, *p* = 0.234, Table S2 right panel). In the multivariate logistic regression, reverse associations between ADHD and the systolic BP (exp(ß) = 0.951, 95%-CI 0.930–0.971, *p* < 0.001) and the diastolic BP (exp(ß) = 0.948, 95%-CI 0.923–0.974, *p* < 0.001) were observed (Table S2 right panel). In the follow-up study, only *n* = 58 participants (0.5%) in the stringent ADHD group and *n* = 73 participants (0.67%) in the control group had available BP measures. Among this small subsample, we could not detect any significant difference in BP between participants in the stringent ADHD and control groups, as shown in Tables S3 and S4.

## 4. Discussion

When children with ADHD are treated with stimulants, they often show an increase of their usually low BP concurrently with symptom improvement [51,52,53,54]. This clinical observation and additional findings from diurnal heart rate, cortisol level and sleep stages suggest a dysregulation of the autonomic nervous system in children with ADHD and leads to the subcortical (low) arousal hypothesis. Hence, in this post-hoc analysis, the naturalistic data from the nationwide representative KiGGS study were used to test the arousal hypothesis with regard to BP regulation in children with ADHD aged between 7 and 17 years. In an earlier cross-sectional study from the KiGGS baseline cohort, ADHD was significantly associated with lower BP [37]. In order to extend and deepen this finding, KiGGS baseline and Wave 2 data from a different age distribution were compared between youngsters with vs. without ADHD including at Wave 2 longitudinal data of a ten-year follow-up assessment, adding a developmental perspective.

Even after broadening the age spectrum to 7 to 17 years, and therefore including a larger sample size (*n* = 9741 controls, *n* = 1219 ADHD) compared to our previous study [37] (*n* = 1219 controls vs. *n* = 667 ADHD), it was shown that systolic and diastolic BP recordings within the ADHD group were significantly below the corresponding values from the non-ADHD control group at baseline, whereas heart rate was similar. In order to improve the comparability of the two groups, we also used an additional statistical tool, namely the propensity score matching technique. The results from this statistical approach demonstrated that systolic and diastolic BP recordings were significantly lower in ADHD patients compared with non-ADHD controls both before and after matching. These findings were observed in both univariate models and multivariate models with age, gender, socioeconomic status, and BMI as confounders and were strengthened by similar results of an additional sensitivity analysis with more stringent ADHD criteria and a smaller ADHD group size (*n* = 272). Thus, according to the arousal hypothesis, the lower BP in ADHD should indicate a subcortical hypoarousal of their brain [26,55]. It is assumed that the symptoms of hyperactivity and sensation seeking, which are typical in ADHD, may represent a behavioral compensatory approach to equalize this state of hypoarousal [28,56,57].

It is noteworthy that the differences between ADHD and controls on systolic and diastolic BP were of high statistical significance (*p* < 0.001), but only of small effect sizes. The slight decrease of effect sizes after matching suggests that findings on autonomic arousal in ADHD, which did not thoroughly consider confounders, should be interpreted with caution.

Significantly low systolic and diastolic BP values for ADHD at baseline could be revealed even after strongly matching the two samples, remaining with small effect sizes of Cohen’s d = 0.17 and Cohen’s d = 0.16, respectively. However, when more stringent ADHD criteria were applied, in a sensitivity analysis the values for Cohen’s d were 0.25 and 0.27, respectively, for a smaller matched sample. Whether this might mean that subcortical hypoarousal is merely expressed in more severe ADHD remains to be investigated. The small effect sizes are not a real surprise for subcortical parameters as can be seen from a large-scale (*n* = 1713 ADHD, *n* = 1529 controls) cross-sectional meta-analysis of magnetic resonance imaging (MRI)-based brain volume differences comparing children and adults with vs. without ADHD. There were significantly smaller volumes in children (below the age of 15 years) with ADHD but not in adults with ADHD. Thus, they extended the brain maturation delay theory for ADHD to include subcortical regions [58]. For their data of children, the authors reported small effect sizes (e.g., nucleus accumbens Cohen’s d = −0.19, amygdala Cohen’s d = −0.18, caudate Cohen’s d = −0.13), probably suggesting that multiple small subcortical deviations within the autonomic nervous system might contribute to the hypoarousal in ADHD. Since brain structure and function are closely related, our findings with a low BP (reflecting low subcortical arousal) in children with ADHD are in line with the view of subcortical neuronal deviations in ADHD. It has to be added that well-grounded models of fronto-striatal dysfunction in ADHD (including deviations of cortical activation patterns, as in Rodríguez et al. [22]) are closely related to cortical structures, which may play a more important role in the severity of ADHD symptoms than the (interacting) subcortical ones [58].

In contrast to the findings within the larger cohort at baseline, within the smaller ten-year follow-up cohort, no significant difference for BP could be observed. This change is not entirely due to the shrinking of the sample size, as we could replicate the association between ADHD and lower BP at baseline measurement within the smaller cohort. The lack of follow-up differences might be due to brain maturational effects. The much lower number of BP values available might have played a minor role, since the similar small sample size of ADHD at baseline still showed up with significant differences of BP. Given the higher age of the participants at follow-up, BMI and BP increased during the ten-year period. Even after checking for confounders in the multivariate logistic regression models, the associations between systolic and diastolic BP and ADHD were no longer significant at follow-up and thus a model of subcortical maturational delay might be applied as in Hoogman et al. [58].

Probably, the increase of the BP values at the follow-up examination of this study may reflect that the hypoarousal hypothesis (valid for children; also verified with other measures of autonomous arousal [30,31,32,33]) no longer holds true for the adolescent and young adult group. An explanation could be the developmental improvement of frontally related compensatory neuronal circuits and/or the recovering of usually more general maturational processes with age in ADHD during late adolescence and young adulthood, at least in cases with a decrease in ADHD symptomatology over time [59,60,61].

A strength of our population study was the large and representative sample size, as well as the possibility of longitudinal observation. Furthermore, propensity score matching enabled a higher comparability of the participants with and without ADHD and an additional sensitivity analysis underlined the robustness of our findings. As a limitation, it must be mentioned that it was not possible to include data on drug treatment of the participants. On the other hand, stimulants, as the standard medication for ADHD, could have increased BP and thus having counteracted our hypothesis. ADHD diagnosis at follow-up was not available. Due to the lack of BP values in the majority of the participants, there was a substantial reduction in sample size at the time of follow-up.

## 5. Conclusions

In summary, significant differences of small effect sizes were found in BP recordings between children with vs. without clinical signs of ADHD, but these differences seem to disappear in young adulthood. Our analysis demonstrated that hemodynamic parameters should be considered to better understand the pathophysiology of ADHD in the developmental context of the subcortical arousal hypothesis.

## Figures and Tables

**Table 1:** Characterization of participants in the ADHD and control group at German Health Interview and Examination Survey for Children and Adolescents (KiGGS) baseline (before and after matching).

Variable	Before Matching			After Matching		
	Participants in the ADHD Group	Participants in the Control Group	*p* Value	Participants in the ADHD Group	Participants in the Control Group	*p* Value
	(*n* = 1219)	(*n* = 9741)		(*n* = 1190)	(*n* = 1190)	
Age (years)	11.25 ± 2.91	11.92 ± 3.13	<0.001	11.26 ± 2.92	11.27 ± 3.07	0.891
Sex (%)						
Female	26.74	51.48	<0.001	26.81	26.64	0.963
SDQ-H	6.99 ± 1.90	2.54 ± 1.79	<0.001	6.97 ± 1.91	2.80± 1.81	<0.001
SES Winkler index	10.32 ± 4.16	11.58 ± 4.33	<0.001	10.33 ± 4.17	10.35 ± 4.17	0.912
SES Winkler Category (%)						
Low	37.17	26.13	<0.001	37.23	36.97	0.907
Medium	46.61	47.62		46.47	46.05	
High	16.22	26.25		16.3	16.97	
BMI (kg/m^2^)	19.28 ± 4.20	19.61 ± 4.00	0.007	19.26 ± 4.19	19.31 ± 3.94	0.8
BMI Category (%)						
Underweight (BMI < 18.5)	50.53	44.09	<0.001	51.01	50	0.809
Normal (18.5 ≤ BMI < 25.0)	38.97	45.92		39.08	40.67	
Overweight (25.0 ≤ BMI < 30.0)	8.04	7.31		8.15	7.9	
Obese (BMI > 30.0)	2.46	2.68		1.76	1.43	
Heart rate (bpm)	78.61 ± 11.73	78.47 ± 11.78	0.683	78.54 ± 23.30	78.26 ±11.63	0.565
Systolic BP (mmHg)	107.71 ± 10.69	110.55 ± 11.22	<0.001	107.6 ± 10.66	109.49 ± 10.89	<0.001
Diastolic BP (mmHg)	64.65 ± 7.52	66.39 ± 7.58	<0.001	64.57 ± 7.48	65.75 ± 7.44	<0.001
Pulse pressure (mmHg)	43.06 ± 7.81	44.15 ±7.92	<0.001	43.03 ± 7.78	43.73 ± 7.89	0.028

Note: Bpm = beats per minute; BMI = body-mass index; mmHg = millimeters of mercury; SDQ-H = hyperactivity-inattention subscale of the Strengths and Difficulties Questionnaire; SES = socioeconomic status; pulse pressure = the difference between systolic blood pressure and diastolic blood pressure.

**Table 2:** Results from unadjusted and adjusted logistic regression models with ADHD as dependent variable in the KiGGS baseline cohort (before and after matching). Unadjusted models used univariate logistic regression. Adjusted models used multivariate logistic regression with systolic, diastolic blood pressure, or pulse pressure as independent variables, adjusted for age, sex, socioeconomic status, body-mass index, and heart rate.

Variable	Before Matching (*n* = 10,960)				After Matching (*n* = 2380)			
	Exp (ß)	95%-CI	Wald	*p* Value	Exp (ß)	95%-CI	Wald	*p* Value
Unadjusted Models								
Age	0.933	0.915–0.952	−6.953	<0.001	0.998	0.972–1.025	−0.137	0.891
Sex	0.344	0.301–0.393	−15.74	<0.001	1.009	0.841–1.209	0.093	0.926
SES	0.932	0.919–0.946	−9.423	<0.001	0.999	0.98–1.018	−0.108	0.914
BMI	0.979	0.964–0.994	−2.674	0.007	0.997	0.978–1.017	−0.253	0.8
Heart rate	1.001	0.996–1.006	0.408	0.683	1.002	0.995–1.009	0.575	0.565
Systolic BP	0.976	0.971–0.982	−8.3	<0.001	0.984	0.976–0.991	−4.233	<0.001
Diastolic BP	0.969	0.962–0.977	−7.539	<0.001	0.979	0.968–0.990	−3.836	<0.001
Pulse pressure	0.982	0.975–0.990	−4.526	<0.001	0.989	0.978–0.999	−2.188	0.029
Adjusted Model for Systolic BP								
Age	0.986	0.960–1.014	−0.967	0.333	1.053	1.015–1.093	2.772	0.006
Sex	0.318	0.277–0.365	−16.329	<0.001	0.955	0.794–1.148	−0.491	0.623
SES	0.93	0.916–0.944	−9.465	<0.001	0.998	0.979–1.018	−0.174	0.862
BMI	1.03	1.011–1.050	3.073	0.002	1.023	0.998–1.049	1.826	0.068
Heart rate	1.004	0.998–1.010	1.252	0.211	1.007	0.999–1.014	1.75	0.08
Systolic BP	0.968	0.961–0.975	−8.259	<0.001	0.971	0.961–0.981	−5.624	<0.001
Adjusted Model for Diastolic BP								
Age	0.961	0.936–0.986	−3.021	0.003	1.027	0.993–1.063	1.534	0.125
Sex	0.333	0.291–0.382	−15.777	<0.001	0.987	0.821–1.186	−0.14	0.888
SES	0.929	0.915–0.943	−9.588	<0.001	0.999	0.979–1.018	−0.136	0.892
BMI	1.011	0.993–1.029	1.168	0.243	1.005	0.982–1.029	0.451	0.652
Heart rate	1.003	0.997–1.009	1.057	0.291	1.006	0.999–1.014	1.646	0.1
Diastolic BP	0.969	0.960–0.978	−6.539	<0.001	0.973	0.961–0.985	−4.368	<0.001
Adjusted Model for Pulse Pressure								
Age	0.948	0.924–0.972	−4.121	<0.001	1.015	0.981–1.049	0.841	0.401
Sex	0.333	0.291–0.382	−15.768	<0.001	0.988	0.823–1.187	−0.128	0.898
SES	0.929	0.916–0.944	−9.481	<0.001	0.999	0.979–1.019	−0.119	0.905
BMI	1.01	0.991–1.029	1.031	0.303	1.005	0.982–1.03	0.448	0.654
Heart rate	0.998	0.993–1.004	−0.656	0.512	1.002	0.995–1.009	0.568	0.57
Pulse pressure	0.983	0.974–0.992	−3.747	<0.001	0.985	0.973–0.997	−2.422	0.016

Abbreviations: BMI = Body-mass index; BP = blood pressure; CI = confidence intervals; SES = socioeconomic status.

**Table 3:** Characterization of matched participants in the ADHD and control group at KiGGS follow-up.

Variable	Participants in the ADHD Group	Participants in the Control Group	*p* Value
	(*n* = 273)	(*n* = 323)	
Measurement at Baseline			
Age (years)	10.60 ± 2.93	10.56 ± 2.99	0.879
Sex (%)			
Female	28.57	25.39	0.435
SDQ-H	6.97 ± 1.93	2.74 ± 1.82	<0.001
SES Winkler index	10.97 ± 3.98	10.65 ± 4.13	0.342
SES Winkler Category (%)			
Low	29.3	35.6	0.252
Medium	50.92	45.51	
High	19.78	18.89	
BMI (kg/m^2^)	18.59 ± 4.11	18.46 ± 3.56	0.684
BMI Category (%)			
Underweight (BMI < 18.5)	61.9	60.06	0.425
Normal (18.5 ≤ BMI < 25.0)	30.77	33.44	
Overweight (25.0 ≤ BMI < 30.0)	5.86	6.19	
Obese (BMI > 30.0)	1.47	0.31	
Heart rate (bpm)	80.00 ± 11.34	78.96 ± 10.93	0.252
Systolic BP (mmHg)	106.14 ± 10.74	107.73 ± 10.69	0.071
Diastolic BP (mmHg)	63.8 ± 7.59	64.99 ± 7.44	0.055
Pulse pressure (mmHg)	42.34 ± 7.32	42.74 ± 7.91	0.519
Measurement at Follow-Up			
Age (years)	21.53 ± 2.92	21.48 ± 3.04	0.856
BMI at (kg/m^2^)	24.81 ± 5.40	23.86 ± 4.02	0.014
BMI Category (%)			
Underweight (BMI < 18.5)	4.76	5.26	0.017
Normal (18.5 ≤ BMI < 25.0)	56.41	60.37	
Overweight (25.0 ≤ BMI < 30.0)	22.34	26.32	
Obese (BMI > 30.0)	16.48	8.05	
Heart rate (bpm)	75.46 ± 12.93	73.55 ± 11.80	0.06
Systolic BP (mmHg)	123.4 ± 10.65	123.78 ± 11.1	0.675
Diastolic BP (mmHg)	71.86 ± 6.84	71.85 ± 7.06	0.992
Pulse pressure (mmHg)	51.55 ± 7.86	51.93 ± 8.05	0.561
Baseline vs. Follow-Up Change			
BMI at (kg/m^2^)	6.22 ± 3.9.0	5.41 ± 3.22	0.006
Heart rate (bpm)	−4.54 ± 13.04	−5.41 ± 13.37	0.427
Systolic BP (mmHg)	17.27 ± 11.44	16.05 ± 12.73	0.223
Diastolic BP (mmHg)	8.05 ± 8.00	6.86 ± 8.65	0.083
Pulse pressure (mmHg)	9.21 ± 9.81	9.19 ± 10.45	0.976

Abbreviations: BMI = body-mass index; BP = blood pressure; bpm = beats per minute; mmHg = millimeters of mercury; SDQ-H = hyperactivity-inattention subscale of the Strengths and Difficulties Questionnaire; SES = socioeconomic status; pulse pressure = the difference between systolic blood pressure and diastolic blood pressure.

**Table 4:** Results from adjusted logistic regression models with ADHD as dependent variable in the matched sample retained at KiGGS follow-up. Adjusted models used multivariate logistic regression with systolic, diastolic blood pressure or pulse pressure at baseline or follow-up as independent variables, adjusted for age, sex, socioeconomic status, as well as body-mass index and heart rate at corresponding time points.

BP at Baseline (*n* =596)					BP at Follow-Up (*n* =596)					Baseline vs. Follow-Up Change (*n* = 596)			
	Exp (ß)	95%-CI	Wald	*p* Value		Exp (ß)	95%-CI	Wald	*p* Value	Exp (ß)	95%-CI	Wald	*p* Value
Adjusted Model for Systolic BP at Baseline or Follow-Up													
Age	1.065	0.987–1.149	1.624	0.104	Age	1.004	0.949–1.063	0.145	0.885	1.024	0.962–1.091	0.749	0.454
Sex	1.118	0.771–1.621	0.586	0.558	Sex	1.065	0.709–1.599	0.302	0.763	1.271	0.853–1.894	1.179	0.239
SES	1.017	0.977–1.060	0.837	0.403	SES	1.027	0.986–1.069	1.261	0.207	1.024	0.983–1.067	1.129	0.259
BMI at baseline	1.039	0.985–1.096	1.4	0.161	BMI at follow-up	1.053	1.015–1.093	2.751	0.006	1.05	1.013–1.090	2.65	0.008
Heart rate at baseline	1.014	0.998–1.030	1.687	0.092	Heart rate at follow-up	1.014	1.000–1.028	1.991	0.046	1.012	0.998–1.026	1.727	0.084
Systolic BP at baseline	0.969	0.949–0.991	−2.83	0.005	Systolic BP at follow-up	0.992	0.975–1.009	−0.959	0.337	1.013	0.997–1.029	1.602	0.109
Adjusted Model for Diastolic BP at Baseline or Follow-Up													
Age	1.046	0.973–1.123	1.221	0.222	Age	1.005	0.949–1.065	0.169	0.865	1.012	0.956–1.072	0.419	0.675
Sex	1.129	0.779–1.635	0.639	0.523	Sex	1.128	0.770–1.652	0.619	0.536	1.17	0.801–1.709	0.813	0.416
SES	1.022	0.981–1.064	1.053	0.292	SES	1.026	0.985–1.069	1.256	0.209	1.026	0.985–1.069	1.221	0.222
BMI at baseline	1.021	0.971–1.074	0.805	0.421	BMI at follow-up	1.05	1.012–1.089	2.629	0.009	1.053	1.016–1.093	2.774	0.006
Heart rate at baseline	1.015	0.999–1.031	1.814	0.07	Heart rate at follow-up	1.014	1.000–1.028	1.956	0.05	1.012	0.999–1.026	1.762	0.078
Diastolic BP at baseline	0.967	0.942–0.992	−2.604	0.009	Diastolic BP at follow-up	0.993	0.968–1.018	−0.572	0.567	1.02	0.999–1.041	1.878	0.06
Adjusted Model for Pulse Pressure at Baseline or Follow-Up													
Age	1.016	0.949–1.088	0.463	0.644	Age	1	0.945–1.058	−0.004	0.997	1.003	0.944–1.065	0.101	0.919
Sex	1.174	0.812–1.696	0.854	0.393	Sex	1.076	0.715–1.619	0.351	0.726	1.16	0.779–1.726	0.731	0.465
SES	1.022	0.981–1.064	1.04	0.299	SES	1.027	0.986–1.069	1.263	0.207	1.026	0.985–1.069	1.241	0.214
BMI at baseline	1.016	0.965–1.070	0.613	0.54	BMI at follow-up	1.054	1.015–1.094	2.732	0.006	1.049	1.013–1.089	2.605	0.009
Heart rate at baseline	1.009	0.994–1.024	1.134	0.257	Heart rate at follow-up	1.013	0.999–1.026	1.818	0.069	1.013	0.999–1.027	1.862	0.063
Pulse pressure at baseline	0.99	0.966–1.015	−0.781	0.435	Pulse pressure at follow-up	0.991	0.968–1.014	−0.801	0.423	1.002	0.984–1.020	0.167	0.868

Abbreviations: see Table 1.

## Data Availability

Raw data were generated at the Robert Koch Institute. Derived data supporting the findings of this study are available from the first or the corresponding author (J.S. and B.W.) on request.

## Supplementary Materials

The following are available online at https://www.mdpi.com/1660-4601/18/4/1864/s1, Table S1: Characterization of participants in the stringent ADHD and control group at KiGGS baseline (before and after matching). Table S2: Results from unadjusted and adjusted logistic regression models with stringent ADHD as dependent variable in the KiGGS baseline cohort (before and after matching). Table S3: Characterization of matched participants in the stringent ADHD and control group at KiGGS follow-up. Table S4: Results from adjusted logistic regression models with stringent ADHD as dependent variable in the matched sample retained at KiGGS follow-up. Figure S1: Distribution of the propensity score for ADHD (before and after matching). Figure S2: Group participants and matching flowchart.
