# Supplementary material for: The Association between Low Blood Pressure and Attention-Deficit Hyperactivity Disorder (ADHD) Observed in Children/Adolescents Does Not Persist into Young Adulthood. A Population-Based Ten-Year Follow-Up Study
Source: Int J Environ Res Public Health. 2021 Feb 14;18(4):1864. doi: 10.3390/ijerph18041864 (PMC7918102; doi:10.3390/ijerph18041864)
Supplement: Supplementary file 1 [file ijerph-18-01864-s001.pdf]

Supplementary Table 1. Characterization of participants in the stringent ADHD and control group at KiGGS baseline (before and after matching).

Variable	Before matching				After matching			
	**Participants in the stringent ADHD group (n=272)**	**Participants in the control group (n=9,741)**	***p*-value**	**Effect size**	**Participants in the stringent ADHD group (n=265)**	**Participants in the control group (n=265)**	***p*-value**	**Effect size**
Age (years)	11.25 ± 2.72	11.92 ± 3.13	<.001	0.214	11.23 ± 2.72	11.29 ± 3.03	.821	0.197
Sex (%)								
Female	19.12	51.48	<.001	0.105	19.25	17.74	.737	0.019
SDQ-H	8.21 ± 1.05	2.54 ± 1.79	<.001	3.205	8.19 ± 1.04	2.89 ± 1.82	<.001	3.576
SES Winkler index	10.36 ± 4.19	11.58 ± 4.33	<.001	0.282	10.35 ± 4.21	10.51 ± 4.20	.680	0.036
SES Winkler category (%)								
Low	36.06	26.13	<.001	0.046	36.23	36.98	.503	0.051
Medium	47.96	47.62			47.55	43.40		
High	15.99	26.25			16.23	19.62		
BMI (kg/m²)	19.31 ± 4.34	19.61 ± 4.00	.238	0.073	19.32 ± 4.36	18.66 ± 3.39	.053	0.168
BMI category (%)								
Underweight (BMI < 18.5)	51.84	44.09	.010	0.034	52.45	53.58	.078	0.113
Normal (18.5 ≤ BMI < 25.0)	35.66	45.92			35.85	40.38		
Overweight (25.0 ≤ BMI < 30.0)	9.19	7.31			9.43	5.66		
Obese (BMI > 30.0)	3.13	2.68			2.26	0.38		
Heart rate (bpm)	79.20 ± 11.53	78.47 ± 11.78	.385	0.053	79.14 ± 11.56	77.62 ± 11.26	.124	0.134
Systolic blood pressure (mmHg)	107.08 ± 9.55	110.55 ± 11.22	<.001	0.309	107.04 ± 9.53	109.85 ± 12.50	.004	0.253
Diastolic blood pressure (mmHg)	64.54 ± 7.00	66.39 ± 7.58	<.001	0.244	64.48 ± 7.01	66.46 ± 7.43	.002	0.273
Pulse pressure (mmHg)	42.54 ± 7.67	44.15 ± 7.92	<.001	0.203	42.55 ± 7.60	43.39 ± 8.49	.023	0.104

Abbreviations: BMI = Body-mass index; BP = blood pressure; bpm = beats per minute; mmHg = millimeters of mercury; SDQ-H = hyperactivity-inattention subscale of the Strengths and Difficulties Questionnaire; SES = socioeconomic status

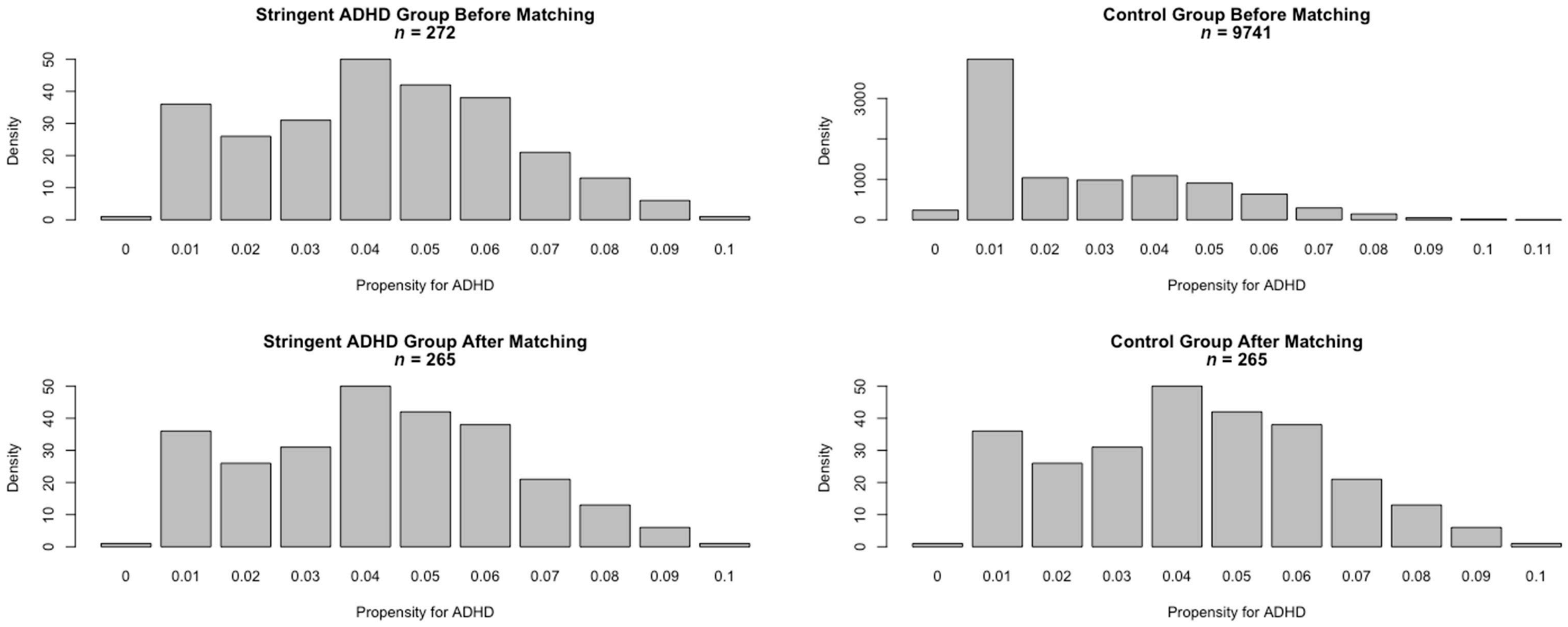

Supplementary Figure 1. Distribution of the propensity score for ADHD (before and after matching).

Supplementary Table 2. Results from unadjusted and adjusted logistic regression models with **stringent** ADHD as dependent variable in the KiGGS baseline cohort (before and after matching). Unadjusted models used univariate logistic regression. Adjusted models used multivariate logistic regression with systolic, diastolic blood pressure or pulse pressure as independent variables, adjusted for age, sex, socioeconomic status, body-mass index, and heart rate.

**Variable**	**Before matching (*n* = 10,960)**				**After matching (*n* = 530)**			
	**Exp (ß)**	**95%-CI**	**Wald**	***p*-value**	Exp (ß)	95%-CI	Wald	*p*-value
Unadjusted models								
Age	0.933	0.897-0.970	-3.467	.001	0.993	0.936-1.054	-0.227	.821
Sex	0.223	0.163-0.300	-9.656	-	1.105	0.713-1.718	0.447	.655
SES	0.934	0.906-0.962	-4.528	-	0.991	0.952-1.033	-0.414	.679
BMI	0.981	0.950-1.012	-1.182	.237	1.044	0.999-1.092	1.921	.055
Heart rate	1.005	0.994-1.015	0.868	.385	1.012	0.997-1.027	1.538	.124
Systolic BP	0.970	0.959-0.982	-4.995	.000	0.977	0.962-0.993	-2.863	.004
Diastolic BP	0.968	0.952-0.983	-3.948	.000	0.963	0.939-0.986	-3.092	.002
Pulse pressure	0.973	0.957-0.989	-3.290	.001	0.987	0.966-1.008	-1.189	.234
Adjusted model for systolic BP								
Age	1.012	0.957-1.070	1.389	.165	1.060	0.977-1.151	1.389	.283
Sex	0.199	0.144-0.269	-10.134	.000	1.020	0.646-1.613	0.086	.932
SES	0.932	0.903-0.960	-4.574	.000	1.002	0.960-1.046	0.096	.924
BMI	1.048	1.009-1.088	2.463	.014	1.111	1.050-1.178	3.602	.000
Heart rate	1.012	1.001-1.024	2.064	.039	1.020	1.003-1.037	2.295	.022
Systolic BP	0.955	0.940-0.970	-5.737	.000	0.951	0.930-0.971	-4.559	.000
Adjusted model for diastolic BP								
Age	0.968	0.919-1.019	-1.244	.214	1.010	0.937-1.089	0.260	.795
Sex	0.213	0.155-0.289	-9.757	.000	1.046	0.664-1.651	0.195	.845
SES	0.930	0.902-0.959	-4.675	.000	1.002	0.960-1.045	0.083	.934
BMI	1.018	0.982-1.055	0.997	.319	1.076	1.021-1.136	2.720	.007
Heart rate	1.010	0.999-1.022	1.170	.082	1.020	1.003-1.037	2.265	.023
Diastolic BP	0.963	0.945-0.981	-3.894	.000	0.948	0.923-0.974	-3.878	.000
Adjusted model for pulse pressure								
Age	0.962	0.913-1.013	-1.478	.139	0.996	0.923-1.075	-0.104	.918
Sex	0.211	0.153-0.285	-9.814	.000	1.066	0.680-1.674	0.279	.781
SES	0.932	0.904-0.960	-4.568	.000	1.003	0.962-1.046	0.143	.886
BMI	1.022	0.984-1.059	1.152	.249	1.079	1.022-1.141	2.724	.006
Heart rate	1.004	0.993-1.016	0.749	.454	1.013	0.997-1.029	1.539	.124
Pulse pressure	0.970	0.952-0.988	-3.235	.001	0.973	0.947-0.999	-2.030	.042

Abbreviations: CI = confidence intervals; Socioeconomic status = SES; BMI = Body-mass index; BP = blood pressure; Pulse pressure = the difference between systolic blood pressure and diastolic blood pressure.

Supplementary Table 3. Characterization of matched participants in the **stringent** ADHD and control group at KiGGS follow-up**.**

Variable	Participants in the stringent ADHD group (*n*=58)	Participants in the control group (*n*=73)	*p*-value	Effect size
**Measurement at baseline**				
Age (years)	10.78 ± 2.63	10.56 ± 2.95	.666	0.076
Sex (%)				
Female	24.14	15.07	.277	0.115
SDQ-H	8.31 ± 1.11	2.82 ± 1.83	<.001	3.534
SES Winkler index	11.62 ± 3.95	10.71 ± 3.85	.187	0.233
SES Winkler category (%)				
Low	25.86	31.51	.610	0.087
Medium	50.00	50.68		
High	24.14	17.81		
BMI (kg/m^2)	17.93 ± 3.51	17.80 ± 3.04	.811	0.042
BMI category (%)				
Underweight (BMI < 18.5)	72.41	63.01	.396	0.151
Normal (18.5 ≤ BMI < 25.0)	22.41	32.88		
Overweight (25.0 ≤ BMI < 30.0)	3.45	4.11		
Obese (BMI > 30.0)	1.72	0.00		
Heart rate (bpm)	79.71 ± 11.25	77.51 ± 10.35	.247	0.205
Systolic blood pressure (mmHg)	105.85 ± 8.70	107.75 ± 11.55	.302	0.182
Diastolic blood pressure (mmHg)	64.97 ± 6.55	66.00 ± 7.21	.401	0.148
Pulse pressure (mmHg)	40.88 ± 6.16	41.75 ± 7.11	.464	0.129
**Measurement at follow-up**				
Age (years)	21.62 ± 2.54	21.48 ± 3.00	.775	0.050
BMI (kg/m^2)	24.56 ± 4.61	23.92 ± 5.08	.455	0.132
BMI category (%)				
Underweight (BMI < 18.5)	3.45	8.22	.458	0.141
Normal (18.5 ≤ BMI < 25.0)	56.90	63.01		
Overweight (25.0 ≤ BMI < 30.0)	25.86	17.81		
Obese (BMI > 30.0)	13.79	10.96		
Heart rate (bpm)	77.02 ± 12.65	75.26 ± 13.20	.442	0.136
Systolic blood pressure (mmHg)	125.61 ± 9.80	126.72 ± 9.26	.508	0.117
Diastolic blood pressure (mmHg)	73.76 ± 6.95	75.31 ± 8.41	.261	0.198
Pulse pressure (mmHg)	51.85 ± 7.85	51.41 ± 7.67	.749	0.056
**Baseline vs follow-up change**				
BMI at (kg/m^2)	6.63 ± 3.70	6.12 ± 4.29	.478	0.125
Heart rate (bpm)	-2.69 ± 12.89	-2.25 ± 12.29	.841	0.035
Systolic blood pressure (mmHg)	19.76 ± 9.62	18.97 ± 12.18	.690	0.070
Diastolic blood pressure (mmHg)	8.79 ± 7.51	9.31 ± 8.79	.720	0.063
Pulse pressure (mmHg)	10.97 ± 9.67	9.66 ± 9.96	.452	0.133

Abbreviations: SDQ-H = hyperactivity-inattention subscale of the Strengths and Difficulties Questionnaire; socioeconomic status = SES; BMI = body-mass index; bpm = beats per minute; mmHg = millimeters of mercury; Pulse pressure = the difference between systolic blood pressure and diastolic blood pressure.

Supplementary Table 4. Results from adjusted logistic regression models with **stringent** ADHD as dependent variable in the matched sample retained at KiGGS follow-up. Adjusted models used multivariate logistic regression with systolic, diastolic blood pressure or pulse pressure at baseline or follow-up as independent variables, adjusted for age, sex, socioeconomic status, as well as body-mass index and heart rate at corresponding time points.

	**Blood pressure at baseline (*n* = 131)**					**Blood pressure at follow-up (*n* = 131)**				**Baseline vs follow-up change (*n* = 131)**			
**Variable**	**Exp (ß)**	**95% CI**	**Wald**	***p*-value**		**Exp (ß)**	**95% CI**	**Wald**	*p*-value	Exp (ß)	95% CI	Wald	*p*-value
Adjusted model for systolic BP at baseline or follow-up													
Age	1.135	0.950-1.366	1.376	.169	Age	1.050	0.921-1.200	0.733	.463	1.078	0.928-1.258	0.978	.328
Sex	1.529	0.599-3.972	0.887	.375	Sex	1.458	0.541-4.007	0.744	.457	1.796	0.695-4.762	1.203	.229
SES	1.079	0.981-1.191	1.553	.120	SES	1.091	0.991-1.206	1.757	.079	1.084	0.985-1.198	1.632	.103
BMI at baseline	1.076	0.925-1.256	0.954	.340	BMI at follow-up	1.054	0.974-1.144	1.292	.020	1.044	1.968-1.130	1.103	.270
Heart rate at baseline	1.033	0.996-1.072	1.719	.086	Heart rate at follow-up	1.020	0.989-1.053	1.221	.222	1.014	0.985-1.046	0.938	.348
Systolic BP at baseline	0.954	0.905-1.002	-1.836	.066	Systolic BP at follow-up	0.982	0.941-1.024	-0.850	.395	1.015	0.977-1.055	0.770	.441
Adjusted model for diastolic BP at baseline or follow-up													
Age	1.089	0.923-1.292	1.009	.313	Age	1.080	0.942-1.244	1.092	.275	1.044	0.915-1.192	0.639	.523
Sex	1.607	0.632-4.159	0.995	.320	Sex	1.518	0.587-4.012	0.858	.391	1.690	0.663-4.407	1.095	.274
SES	1.087	0.989-1.201	1.702	.089	SES	1.093	0.993-1.209	1.781	.075	1.087	0.988-1.200	1.683	.092
BMI at baseline	1.040	0.901-1.197	0.544	.586	BMI at follow-up	1.051	0.973-1.138	1.258	.208	1.043	0.967-1.127	1.086	.278
Heart rate at baseline	1.031	0.995-1.071	1.671	.095	Heart rate at follow-up	1.027	0.994-1.062	1.577	.115	1.017	0.987-1.049	1.089	.276
Diastolic BP at baseline	0.958	0.900-1.017	-1.397	.162	Diastolic BP at follow-up	0.957	0.906-1.007	-1.647	.100	0.990	0.946-1.035	-0.432	.666
Adjusted model for pulse pressure at baseline or follow-up													
Age	1.087	0.917-1.294	0.953	.341	Age	1.056	0.924-1.209	0.798	.425	1.097	0.945-1.282	1.195	.232
Sex	1.721	0.687-4.411	1.154	.248	Sex	1.843	0.695-5.044	1.220	.223	1.909	0.731-5.144	1.311	.190
SES	1.073	0.976-1.183	1.441	.150	SES	1.086	0.987-1.200	1.667	.095	1.080	0.980-1.194	1.539	.124
BMI at baseline	1.039	0.898-1.202	0.529	.597	BMI at follow-up	1.037	0.959-1.123	0.914	.361	1.042	0.965-1.127	1.048	.295
Heart rate at baseline	1.026	0.990-1.064	1.400	.161	Heart rate at follow-up	1.016	0.987-1.048	1.061	.289	1.017	0.987-1.048	1.094	.274
Pulse pressure at baseline	0.966	0.901-1.035	-0.975	.329	Pulse pressure at follow-up	1.016	0.967-1.069	0.629	.529	1.028	0.985-1.074	0.244	.214

Note. CI = confidence intervals; SES = socioeconomic status; BMI = body-mass index; BP = blood pressure; pulse pressure = the difference between systolic blood pressure and diastolic blood pressure

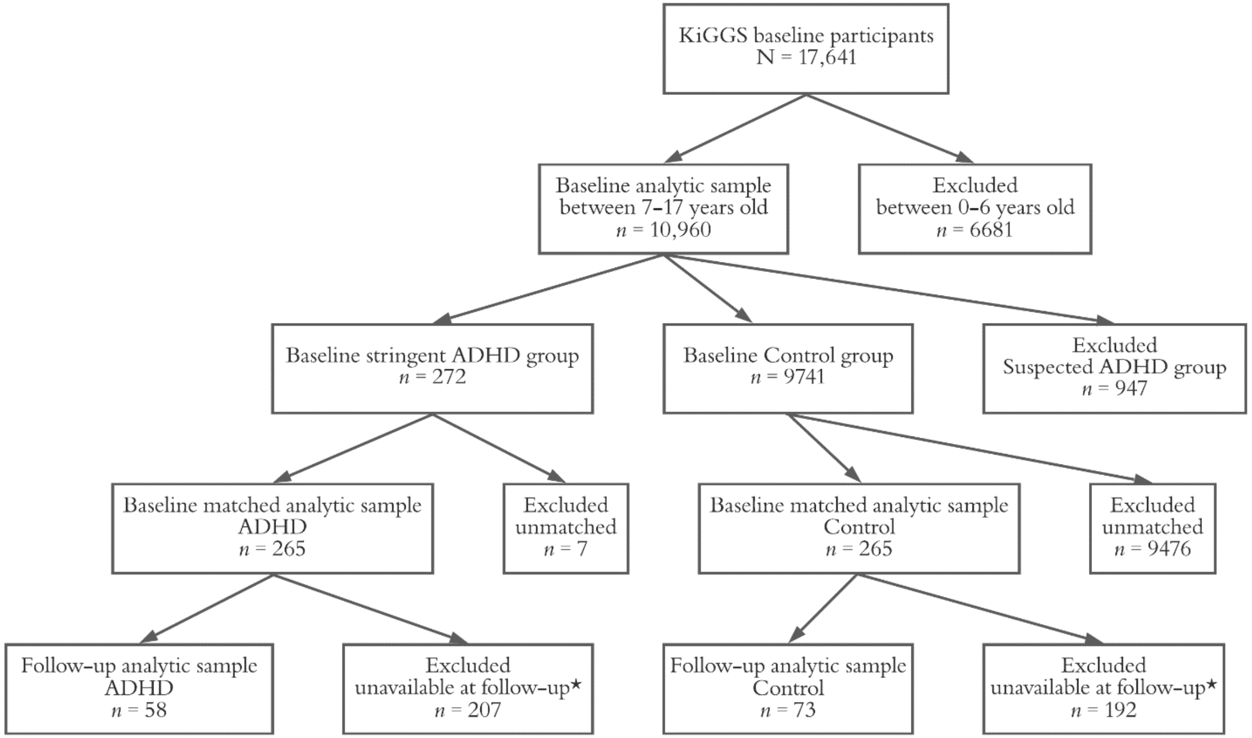

Supplementary Figure 2. Group participants and matching flowchart. **Note.** *Participants were excluded due to lack of physical measurements of blood pressure at the ten-year follow up.
